# Giant Cerebral Hydatid Cyst: A Rare Case Report

**DOI:** 10.1002/ccr3.3908

**Published:** 2021-02-10

**Authors:** Babak Ganjeifar, Majid Ghafouri, Azar Shokri, Farhad Rahbarian Yazdi, Seyed Ahmad Hashemi

**Affiliations:** ^1^ Department of Neurosurgery Mashhad University of Medical Sciences Mashhad Iran; ^2^ Vector‐borne Disease Research Center North Khorasan University of Medical Sciences Bojnurd Iran; ^3^ Department of Neurosurgery North Khorasan University of Medical Sciences Bojnurd Iran

**Keywords:** brain, hydatid cyst, Iran, surgery

## Abstract

The diagnosis of hydatid cyst should be considered in children with seizure in endemic regions.

## Graphical abstract

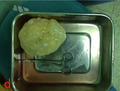

## 1 INTRODUCTION

Here we describe a 13‐year‐old patient with the presentation of fever and abdominal pain. He had a history of 2 years headache and seizure. In MRI and computed tomography (CT) scan, a primary cerebral hydatid cyst was evident. The diagnosis of hydatid cyst should be considered in children with mentioned characters in endemic regions.

Hydatid disease, an important zoonotic disease, is caused by infestation of the larva of dog tapeworm *Echinococcus granulosus*.

Dogs are definite hosts for the parasite and pass eggs with their faces. Humans are accidentally infected through ingestion of food or water contaminated with dog faces or by direct contact with dogs.^1^, ^2^ The disease is common in the Mediterranean countries, the Middle East, Australia, and New Zealand.^3^ Cysts occur most commonly in the liver or lungs.^1^ The intracranial involvement is rare and with the incidence rate 1%‐2% of all hydatidosis cases.^4^ We describe a case of a giant cerebral hydatid cyst in a 13‐year‐old boy.

## 2 CASE REPORT

A 13‐year‐old boy presented to our hospital with abdominal pain and fever. The pain was constant, nonradiation, worsened with light activity. He has any other gastrointestinal symptoms. The patient had no history of previous hospitalization and did not take any medication. The patient lived in a rural zone. In admitting time, the patient was febrile (body temperature 40°C), blood pressure 110/75 mm Hg, heart rate 92 beats/min, and breathing rate 18 bpm. A general physical examination revealed no abnormality.

## 3 DIFFERENTIAL DIAGNOSIS, INVESTIGATIONS, AND TREATMENT

The complete blood investigation performed with the following results: white blood cell count 7400 cell/mm^3^; hemoglobin 12.4 g/dL; and platelets 275 000 cells/mm^3^. Laboratory examinations revealed alkaline phosphatase levels (548 IU/L) which was high. Also, blood and urine culture, serological tests, and C‐reactive protein (CRP) tests were performed. The results of all tests were negative. Urinalysis and other laboratory work‐ups were normal. Erythrocyte sedimentation rate (ESR) was 16 mm/h. Chest radiography, electrocardiogram, and ultrasonography (US) of the abdomen and pelvic were normal.

**FIGURE 1:**
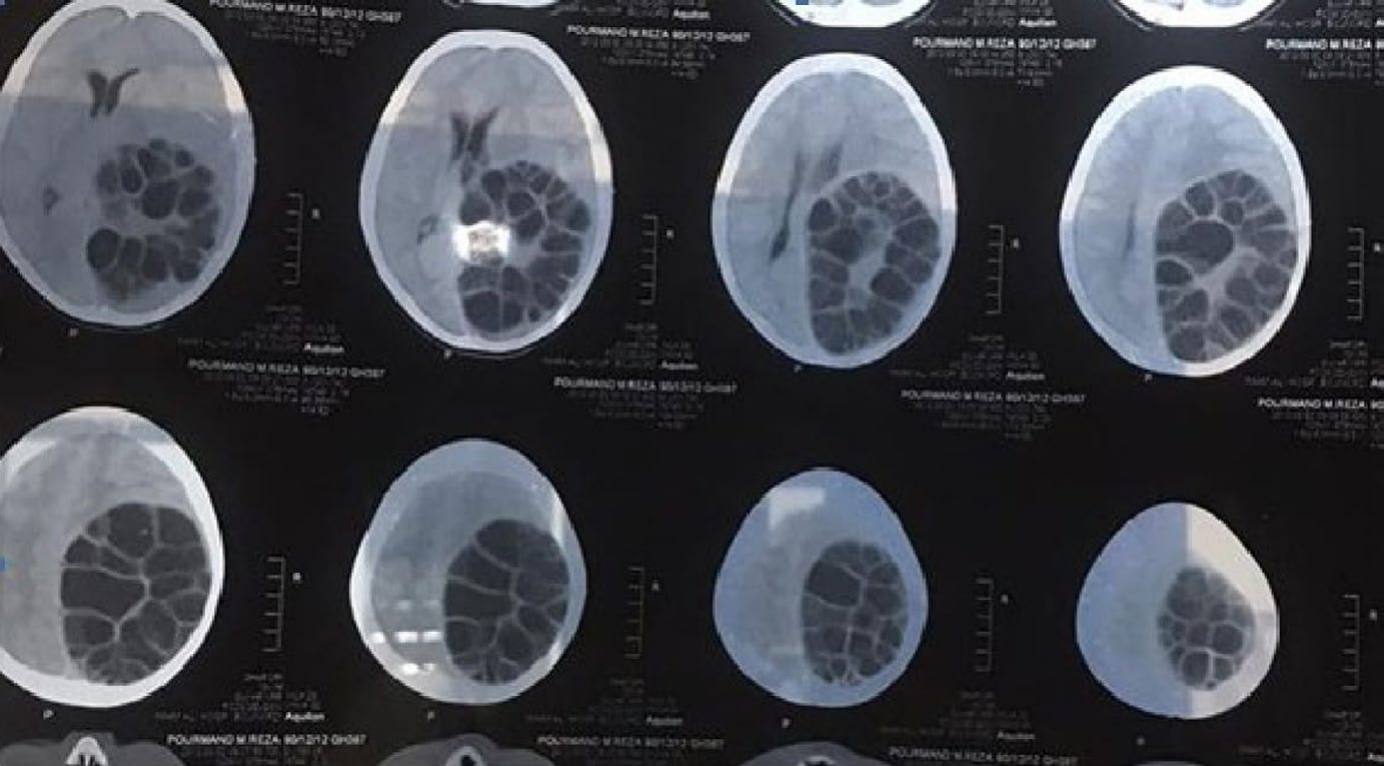
Preoperation CT scan revealed a large cystic left parietal and occipital lobe, paraventricular area

**FIGURE 2:**
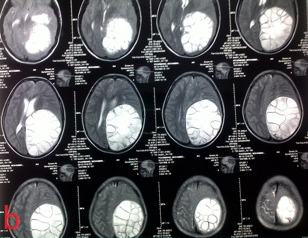
T2‐weighted axial MRI of the brain shows a large cystic left parietooccipital lobe, paraventricular area

Cranial CT revealed a left parieto‐occipital hypodense, cystic, well‐ demarcated (Figure 1). Magnetic resonance imaging (MRI) showed the mass containing membranous structures and with cerebrospinal fluid (CSF) intensity in the left hemisphere, paraventricular area (Figure 2).

**FIGURE 3:**
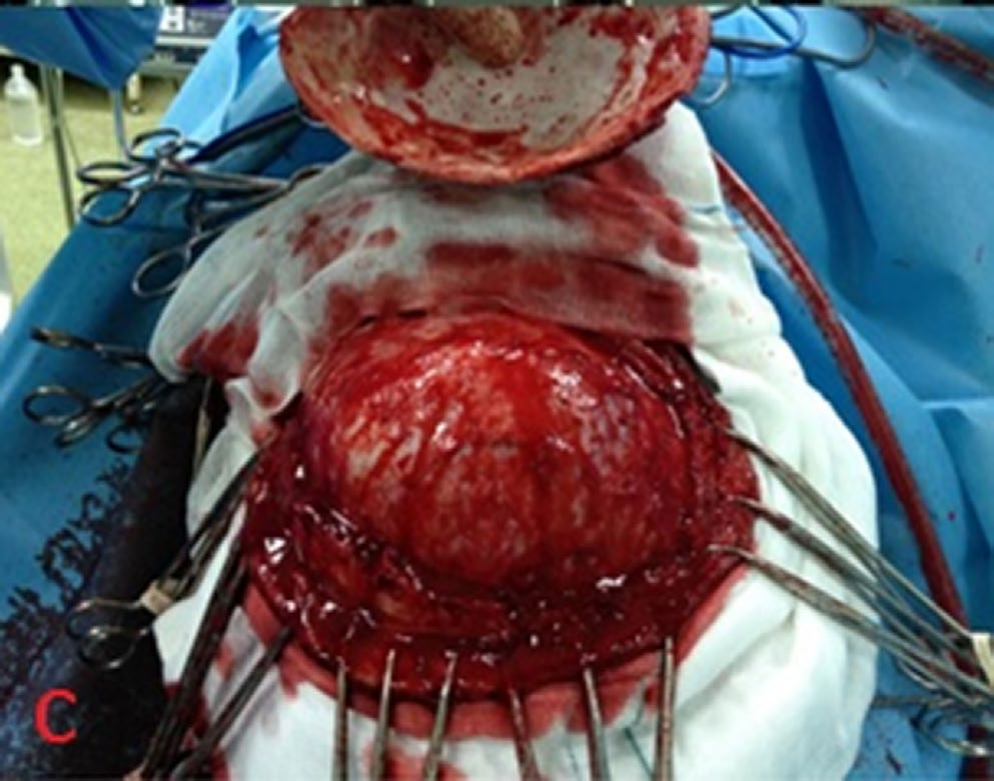
After opening the dura, an intracranial cystic mass was determined. The lesion was removed gross totally

**FIGURE 4:**
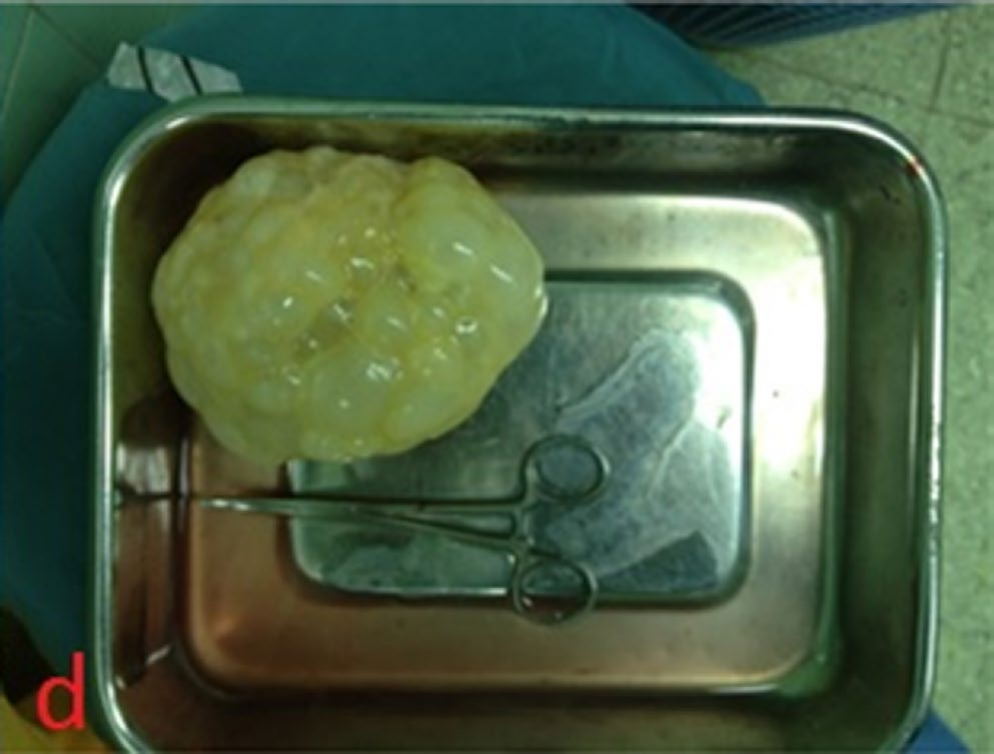
The cyst removed in toto after operation. The cyst appears with creamy and germination of daughter cysts

Based on the cranial imaging findings, differential diagnosis of other cystic lesions such as abscesses, large granulomas, cystic gliomas, epidermal cysts, and arachnoid cysts, arising from the brain were considered. However, imaging and serological findings confirmed the diagnosis of hydatid cyst. A wide parieto‐occipital craniotomy was performed. The dura was dissected (Figure 3) and the cyst expelled without rupture (Figure 4). The patient recovered well after the Surgery. Pathological examination of the specimen confirmed the diagnosis of hydatid cyst.

**FIGURE 5:**
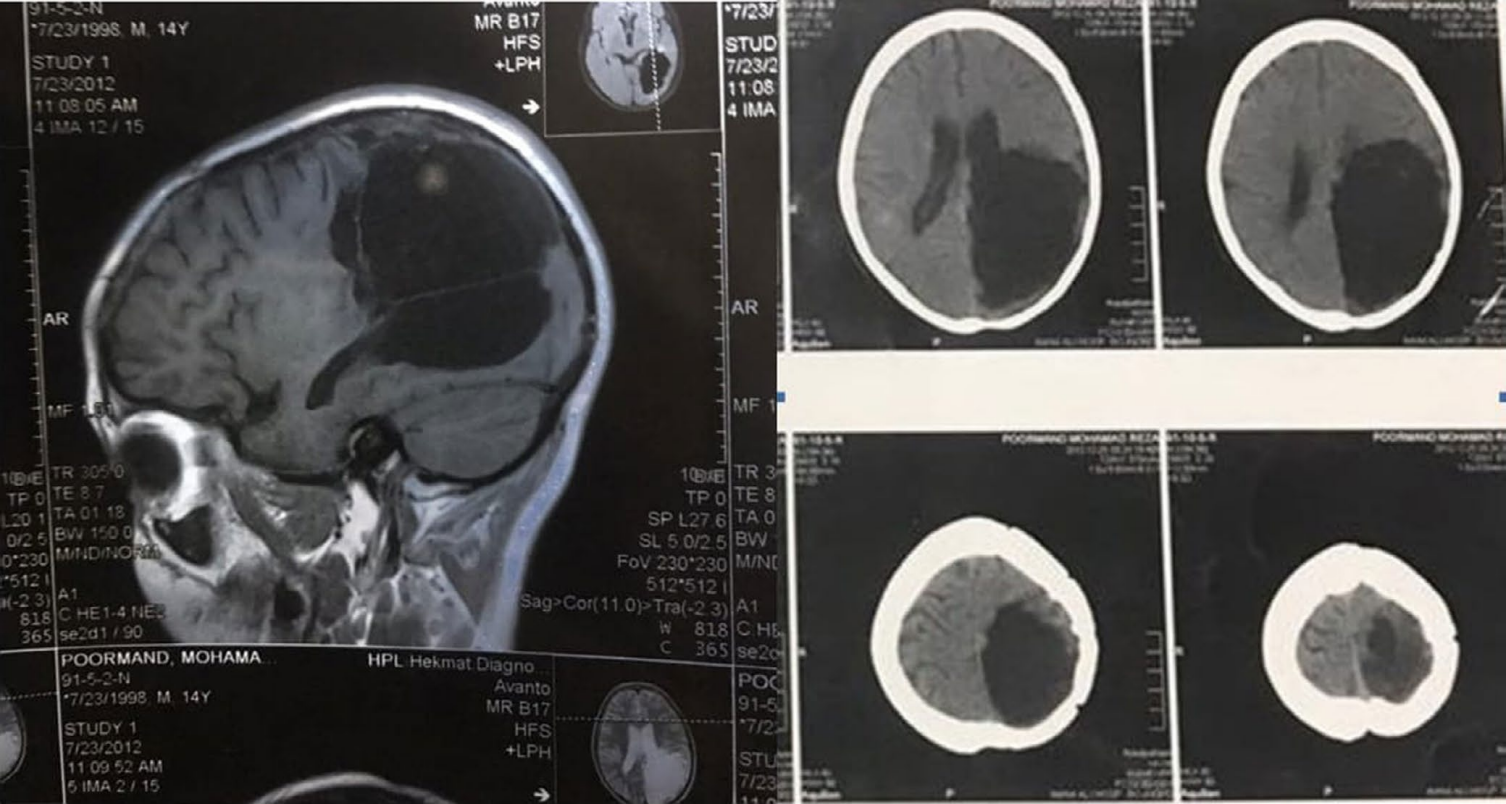
Postoperation follow‐up CT (right) and MRI (left) images shows post‐op changes with surrounding edema that reveals extraction of cystic lesions

Treatment with albendazole was started when the patient was discharged from the hospital for prevention of further echinococcosis, and also, he received Dilantin (phenytoin sodium) (5 mg/kg/d in divided doses) for prevention of seizure. Postoperation MRI and CT scan images revealed no sign of mass in the operated area (Figure 5). The patient fallowed up 3 years after the surgery. Final check up carried out recently. The patient is 18 years old now and decided to deployment to military service. The patient had no symptom and complaints of previous disease.

## 4 DISCUSSION

Echinococcosis is a parasitic disease caused by the larval stage of a tapeworm *E granulosus*. Dogs and other canids are definitive hosts and sheep, goat and other herbivores are intermediate hosts. Humans get infected accidentally through oral‐fecal route by consumption of vegetables or water contaminated with parasite eggs or via direct contact with an infected dog. There are four species of this genus can infect humans: *E granulosus* (causing cystic echinococcosis), *Echinococcus multilocularis* (causing alveolar echinococcosis), *Echinococcus oligarthus*, and *Echinococcus vogeli* (which cause polycystic hydatid disease). After ingestion of eggs, they hatch in the small intestine submucosa and enter veins or lymphatic vessels^5^ and then disseminate into organs such as liver, lung, and other organs. The most common sites are the liver (75%) and lungs (15%) followed by the spleen, kidney, heart, bones, and brain (10%).

Central nervous system hydatidosis is rare and is usually diagnosed during childhood.^6^ In our report, the patient was young and had no disease; living in a rural area and contact with dogs was his only risk factor. Considering hydatid cyst in individual with fever and history of contact with dogs is necessary in endemic areas.

Brain hydatid cyst also is classified as primary (single) or secondary (multiple). The primary cysts are formed as a result of direct infestation of the brain without involvement of other organs and the secondary multiple cysts result from spontaneous, traumatic, or surgical rupture of a solitary cranial cyst.^7^ In the present case, cysts identified only in the brain, so the brain was a primary focus for hydatid cyst.

Hydatid cyst see in anywhere of the brain and most commonly located supratentorially, in the middle cerebral artery territory.^6^ The presented case had a single supratentorially cyst in the left parietal and occipital lobe, and paraventricular area (Figures 1, 2, 3, 4).

Intracranial hydatid cysts are usually present with headache, vomiting, and seizure due to raised intracranial pressure and brain compression. In physical examination, papilledema and neurological deficit may be presented.^8^ This case only presented with generalized abdominal pain, fever, and a history of seizure plus headache. In general examination, no abnormality was found. A combination of tools including imaging techniques and serology are necessary for diagnosis of a patient with hydatidosis. For diagnosis of cystic echinococcosis (CE), imaging techniques (CT scan and MRI) are the most reliable methods while serological tests which detect the specific antigens of *E granulosus* are used for verifying the imaging results.^9^ In our patient, hydatid serological test and CRP were negative and the other laboratory tests were normal.

Computed tomography scan of cranial hydatid cysts showed an intraparenchymal spherical cystic lesion with distinct borders. Also, the cyst fluid was isodense with CSF. MRI imaging showed a low signal intensity rim of the cyst wall, while the cyst content had signal intensities similar to CSF.^10^

In our case, CT scan revealed a left parieto‐occipital hypodense, cystic, well‐demarcated, round lesion causing shift to the right. MRI imaging showed a mass consists of membranous structures with CSF intensity in the paraventricular area of left hemisphere.

Although in small or inoperable brain cysts, medical therapy has shown promising effects but surgery remains the golden treatment by which cysts can be removed without rupture and results in a complete cure. Chemotherapy with two benzimidazoles (ABZ or MBZ) is indicated for inoperable patients with primary liver/lung echinococcosis and for patients with multiple cysts in two or more organs.^11^, ^12^

Dowling technique is the most effective surgical method for removing cerebral hydatid cysts without causing rupture.^13^ For Dowling‐Orlando technique, head puts lower than the operation table. Large craniotomy flap can be made depending on the size and site of the lesion. Surgery area of brain covering cotton soaked with warm normal saline to prevent spillage in case of rupture. Then, the cyst removes and dura closing watertight, bone flap puts back and patient dresses after wound closure.^2^, ^14^ After craniotomy and prior to the incision, the surgical field must be cleaned with scolicidal solution to prevent recurrence because even a minimal spillage can lead to new cysts formation (1 mL of cyst fluid contains 4 000 000 scolices).^15^, ^16^

Albendazole has been used successfully for treatment of hydatid cyst in brain,^17^ for prevention of secondary hydatid disease. Use of benzimidazoles (ABZ or MBZ) before surgery can reduce the risk of recurrence of CE. Chemotherapy is more effective among younger patients. For treatment of CE, oral dosage of 10‐15 mg/kg/d of Albendazole in a course of 1‐month separated by 14‐day interval can be prescribed. Three courses are routinely suggested, and more than six usually will be unnecessary. The usual oral dosage of mebendazole is 40‐50 mg/kg/d for at least 3‐6 months.^18^

In summary, intracranial hydatidosis is rare and more affects pediatric age group. It may misdiagnose as intracranial cyst, so in differential diagnosis of intracranial cyst especially in endemic areas, age of the patients will be helpful. Total surgical remove of the cysts without rupture is still the treatment of choice in cerebral hydatidosis.

## CONFLICT OF INTEREST

None declared.

## AUTHOR CONTRIBUTION

All authors, MGh, ASh, FRY, and SAH, are equally contributed to the design, analysis, and presentation and critically revise of this study. BG: is surgeon of patient. MGh: is specialist in infectious disease and involved in study design. ASh: involved in study design, writing, submission, and revision.

## ETHICS APPROVAL AND CONSENT TO PARTICIPATE

Applicable.

## CONSENT FOR PUBLICATION

Written informed consent was obtained from the parents of patient for publication of this case report and any accompanying images. A copy of the written consent is available for review by the Editor of this journal.

## Data Availability

All the data are available without restriction.
